# Calculation of the Electrical Conductivity of Polymer Nanocomposites Assuming the Interphase Layer Surrounding Carbon Nanotubes

**DOI:** 10.3390/polym12020404

**Published:** 2020-02-11

**Authors:** Yasser Zare, Kyong Yop Rhee

**Affiliations:** Department of Mechanical Engineering, College of Engineering, Kyung Hee University, Yongin 446-701, Korea; y.zare@aut.ac.ir

**Keywords:** polymer/CNT nanocomposites, percolation threshold, interphase, waviness, electrical conductivity

## Abstract

The interphase layer surrounding nanoparticles can reflect the tunneling effect as the main mechanism of charge transferring in polymer/carbon nanotube (CNT) nanocomposites (PCNT). In this paper, the percolation threshold, effective volume fraction of CNT, and the portion of percolated filler after percolation are expressed by interphase and CNT waviness. Moreover, the developed terms are used to suggest the influences of CNT dimensions, interphase thickness, and waviness on the electrical conductivity of PCNT by conventional and developed models. Thin and long CNT, thick interphase, and low waviness obtain a high fraction of percolated CNT. However, the highest level of effective filler fraction is only calculated by the thinnest CNT and the thickest interphase. Furthermore, both models show that the thinnest and the longest CNT as well as the thickest interphase and the least CNT waviness cause the highest conductivity in PCNT, because they positively contribute to the formation and properties of the conductive network.

## 1. Introduction 

The addition of conductive fillers such as carbon nanotubes (CNT) and graphene can increase the electrical conductivity of polymers. The resulting materials as polymer nanocomposites can be used in sensors and electromagnetic interference shielding for electronic tools and electrostatic dissipation [1,2,3,4,5,6,7,8,9,10,11,12,13,14,15,16,17,18,19,20,21,22,23,24,25,26,27]. The typical conductivity of polymers ranges from 10^−12^ to 10^−15^ Siemens/m (S/m), while the electrical conductivity of CNT is in order of 10^5^ to 10^7^ S/m. So, at a critical filler concentration called percolation threshold, the electrical conductivity increases several orders of magnitude and after that, the conductivity levels off close to that of nanofiller [28,29]. At percolation threshold, the filler can begin to form a continuous conductive network in nanocomposite. The percolation level can be experimentally estimated by measurement of electrical conductivity of polymer CNT nanocomposites (PCNT) at dissimilar CNT concentrations. The shape of nanofiller can affect the percolation threshold of nanoparticles and conductivity of nanocomposites. For spherical nanoparticles, a smaller size decreases the percolation threshold [30], while for layered and cylindrical fillers, a larger aspect ratio (length per diameter) lowers the percolation threshold [31]. 

The main difficulty for creating the conductive nanocomposites with a low CNT content is the poor distribution of CNT, due to the tendency of nanotubes to form bundles in which nanotubes stick together with each other due to the van der Waals interaction [32]. The nanotubes combined into bundles do not participate in the percolation chains, which increases the percolation threshold. However, the experimental results show that an absolutely uniform distribution of the additive in the material is not optimal, but the percolation threshold depends on the duration of the heat treatment [33]. In fact, the establishment of a percolation conductive chain in a nanocomposite requires a considerable time during which the filler particles are distributed over the volume of the polymer matrix [33]. 

Some models were proposed to explain and predict the conductivity of polymer composites. The electrical conductivity was commonly characterized by its dependence on filler concentration, because the conductivity of composites is close to that of pure polymer matrix at low filler fractions. Some models also considered the percolation threshold of filler. A known power-law model based on conventional percolation theory was suggested, which has been widely applied for electrical conductivity of PCNT after percolation threshold [30,34,35]. However, this model was developed for conventional composites, and disregards the physical aspects of nanofillers such as nano-size and large surface area per weight. Monte Carlo simulations were also used to analyze the percolation threshold [36], but it’s an expensive method, which does not express clear formulations. 

The interphase as a different phase in polymer nanocomposites is commonly formed due to the outstanding surface area of nanoparticles, which causes strong interfacial interaction between polymer matrix and nanoparticles [37,38]. It was reported that the interphase regions positively affect the mechanical properties of polymer nanocomposites [39,40]. Therefore, many conventional models for mechanical performances of composites have been developed to consider the role of interphase [41,42]. In addition, it was found that the interphase regions usually diminish the percolation threshold of nanoparticles in polymer nanocomposites [43,44,45,46,47], because the interphase layer surrounding the nanoparticles can form a continuous network before the connection of particles. The influences of interphase percolation on the mechanical behavior of PCNT were investigated in the previous reports [44,48], while its role in electrical conductivity has not been deliberated. It seems that the interphase around nanoparticles can play the tunneling effect, which controls the electrical conductivity of PCNT. The main mechanism for charge transfer in PCNT was stated as electron tunneling, where all nanotubes are electrically connected, and electrons are transferred by the tunneling effect [49]. In other words, the near nanoparticles at a determinate tunneling distance produce the conductivity by electron hopping, while the nanotubes are not bodily linked. The tunneling mechanism depends on the distance between nanotubes, while the electrical conductivity of PCNT is only considered by networking of CNT above percolation threshold. 

Some authors developed micromechanics models for electrical conductivity of PCNT accounting the waviness of CNT as well as tunneling distance [50,51], but they did not consider the formation of interphase in nanocomposites. Additionally, the roles of interphase region and CNT waviness in percolation threshold and other effective properties of nanoparticles have not been clearly reported. In this study, the influences of interphase and waviness on percolation threshold, effective volume fraction of CNT and the fraction of percolated (networked) CNT after percolation are plotted and discussed. In addition, these developed terms are applied to evaluate the electrical conductivity of PCNT by conventional and developed models. Actually, this paper clarifies the effects of CNT dimensions, interphase thickness and waviness of CNT on the levels of percolation, effective volume fraction of CNT, percolated CNT and electrical conductivity of PCNT. The present explanations may justify the very low percolation level of CNT as well as the extraordinary conductivity of PCNT, which cannot be obtained by conventional theories. 

## 2. Theoretical Analysis

The percolation threshold for random distribution of CNT in PCNT can be expressed [52] by:

(1) $$\varphi_{p}=\frac{\pi R^{2}l+(4/3)\pi R^{3}}{\frac{32}{3}\pi {\left(R+t\right)}^{3}[1+\frac{3}{4}(\frac{l/u}{R+t})+\frac{3}{32}{\left(\frac{l/u}{R+t}\right)}^{2}]}$$

where “R” and “l” denote the radius and length of straight CNT, correspondingly and “t” shows the interphase thickness. Also, “u” is waviness parameter.

The waviness of CNT in PCNT commonly occurs, which decreases their effectiveness’ for conductivity. An equivalent tube with “l_eq_” length can be regarded by waviness. A waviness parameter is defined as the length of straight nanotubes per equivalent length as:

(2) $$u=\frac{l}{l_{eq}}$$

where u = 1 shows the straight CNT (no waviness), but a higher “u” demonstrates the extent of waviness. 

The CNT and surrounding interphase can be assumed as effective particles, which manage the electrical conductivity of PCNT. The effective volume fraction of straight CNT is expressed [52] by:

(3) $$\varphi_{eff}=\frac{{\left(R+t\right)}^{2}(l+2t)}{R^{2}l}\varphi_{f}$$

where “$\varphi_{f}^{}$” is filler volume fraction.

When the waviness of CNT is considered by the length of equivalent nanotubes, Equation (3) is modified to:

(4) $$\varphi_{eff}=\frac{{\left(R+t\right)}^{2}(l/u+2t)}{R^{2}l/u}\varphi_{f}≅\frac{{\left(R+t\right)}^{2}}{R^{2}}\varphi_{f}$$

Additionally, a number of nanotubes in PCNT create the conductive network after percolation point and others are still dispersed in polymer matrix. An equation was proposed to calculate the fraction of percolated CNT [52] as:

(5) $$f=\frac{\varphi_{f}^{1/3}-\varphi_{p}^{1/3}}{1-\varphi_{p}^{1/3}}$$

When the influences of interphase and CNT waviness on percolation threshold and effective CNT concentration are considered in the above equation, it develops to the following form:

(6) $$f_{iu}=\frac{\varphi_{eff}^{1/3}-\varphi_{p}^{1/3}}{1-\varphi_{p}^{1/3}}$$

All these parameters were mentioned in [52], but the used model is different from the current study. Generally, there are similar models in the literature, which properly fit to the experimental data of real nanocomposite samples [52,53,54]. 

Now, two conventional and developed models are suggested to investigate the effects of interphase and CNT waviness on the electrical conductivity of PCNT. 

A conventional power-law model for electrical conductivity of composites above percolation threshold was suggested [55] as:

(7) $$\sigma =\sigma_{N}{\left(\varphi_{f}-\varphi_{p}\right)}^{b}$$

where “σ_N_” is CNT conduction and “b” is an exponent. The theoretical and experimental studies reported the “b” values in the 1.3–3.1 range [55]. By fitting the experimental levels of electrical conductivity to above equation, “$\varphi_{p}^{}$” and “b” values can be assessed, as reported in many studies [30,34,35].

The previous studies have indicated that the waviness decreases the conductivity of CNT [56]. Accordingly, “σ_N_” parameter can be expressed by:

(8) $$\sigma_{Nu}=\frac{\sigma_{N}}{u}$$

So, Equation (7) can be developed by substituting of Equations (8), (4) and (1) for CNT conductivity, effective filler fraction and percolation threshold, respectively as:

(9) $$\sigma =\sigma_{Nu}{\left(\varphi_{eff}-\varphi_{p}\right)}^{b}$$

which enables the classical model to estimate the electrical conductivity of PCNT. 

Deng and Zheng [51] also suggested a model for electrical conductivity of PCNT above percolation containing haphazardly straight CNT as:

(10) $$\sigma =\sigma_{0}+\frac{f\varphi_{f}\sigma_{N}}{3}$$

where “σ_0_” as the electrical conductivity of polymer matrix can be disregarded, due to its very low level. This model has expressed the proper predictions for electrical conductivity of PCNT [50,51]. When the interphase and waviness of CNT are taken into account by “f_iu_”, “$\varphi_{piu}^{}$” and “σ_Nu_” terms, this model is developed to:

(11) $$\sigma =\frac{f_{iu}\varphi_{eff}\sigma_{Nu}}{3}$$

The tunneling properties can be assumed in the developed model by a tunneling parameter (T) as:

(12) $$\sigma =\frac{Tf_{iu}\varphi_{eff}\sigma_{Nu}}{3}$$

which can easily calculate the electrical conductivity of PCNT by the properties of CNT, interphase, tunneling space and network. The nanocomposites containing all types of polymers (thermoplastics to elastic) and CNT (MWCNT, SWCNT or DWCNT or modified ones) can fit to this equation. Also, this model accurately fits to the experimental data of samples containing low CNT concentrations, because a high filler concentration increases the agglomerates [57]. 

## 3. Results and Discussion

**Figure 1:**
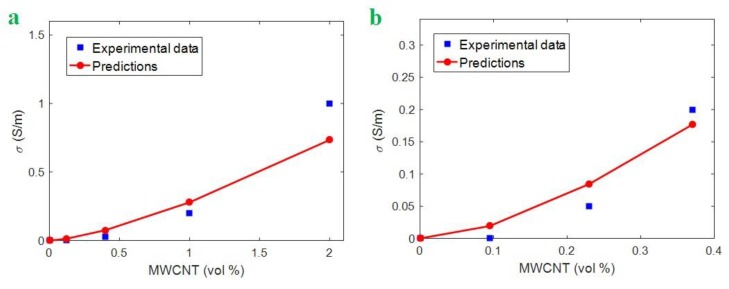
Comparison between experimental and theoretical data (Equation (12)) for (**a**) epoxy/MWCNT [55] and (**b**) UPE/MWCNT [58] samples.

The calculations of the developed model (Equation (12)) are compared to the experimental data of some samples. Two samples including epoxy/MWCNT (R = 8 nm, l = 30 μm, u = 1.2 and $\varphi_{p}^{}$= 0.0002) from [55] and ultrahigh molecular weight polyethylene (UPE)/MWCNT (R = 8 nm, l = 8 μm, u =1.2 and $\varphi_{p}^{}$= 0.0007) from [58] are considered. When the percolation threshold of samples is fitted to Equation (1), the interphase thickness is calculated as 7 and 8 nm for epoxy/MWCNT and UPE/MWCNT samples, respectively. When these results are considered in Equation (12), the conductivity of the samples at different CNT concentrations is calculated (σ_N_ = 10^6^ S/m). Figure 1 displays the comparison between experimental and theoretical data using Eq. 12. It is observed that the developed model acceptably estimates the conductivity for the samples. Therefore, the developed model is capable to estimate the conductivity for the real samples. “T” is also calculated as 0.0001 and 0.00025 for epoxy/MWCNT and UPE/MWCNT samples, respectively. These results indicate that the UPE/MWCNT sample has better tunneling properties compared to the epoxy/MWCNT nanocomposite. 

The influences of different parameters on the percolation threshold, effective volume fraction of CNT, the fraction of percolated filler and electrical conductivity are plotted and discussed using the mentioned equations. Contour plots show the roles of two parameters in an output at average values of other parameters. Generally, the average levels of $\varphi_{f}^{}$= 0.02, R = 10 nm, l = 10 μm, σ_N_ = 10^6^ S/m, t = 4 nm, u = 1.25 and T = 1 are considered for all calculations. 

**Figure 2:**
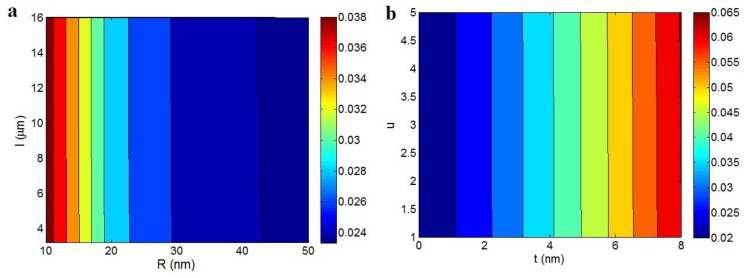
“$\varphi_{eff}^{}$” (Equation (4)) as a function of (**a**) “R” and “l” and (**b**) “t” and “u” factors.

Figure 2 illustrates “$\varphi_{eff}^{}$” levels at different values of “R”, “l”, “t” and “u” parameters using Equation (4). According to Figure 2a, “$\varphi_{eff}^{}$” only depends on “R” parameter and “l” cannot affect it. The highest “$\varphi_{eff}^{}$” is obtained by the least “R”, while “$\varphi_{eff}^{}$” decreases by increment of “R”. The highest “$\varphi_{eff}^{}$” as 0.038 is obtained at R = 10 nm, but the higher “R” (R > 44 nm) produce the least $\varphi_{eff}^{}$< 0.024. Therefore, thin CNT cause positive effect on “$\varphi_{eff}^{}$” at the different levels of “l”. As known, the thin CNT introduce optimistic effects on the general properties of PCNT. Moreover, it was reported that the thin nanoparticles can produce a high level of interphase fraction in nanocomposites [59,60]. So, the thin CNT can more effectively improve the performances of nanocomposites like conductivity, due to their big surface area. However, the independence of “$\varphi_{eff}^{}$” to CNT length can be attributed to its higher levels compared to “R” and “t” parameters. 

Figure 2b also demonstrates the strong effect of “t” parameter on “$\varphi_{eff}^{}$”, while “u” waviness parameter does not play a role. The highest and the lowest “$\varphi_{eff}^{}$” are calculated by the thickest and the thinnest interphase, respectively. Therefore, a thick interphase shows positive role in “$\varphi_{eff}^{}$” term. It is clear that a high level of “t” increases the volume of effective cylinder, which occupies a high effective volume in PCNT. Normally, a high level “$\varphi_{eff}^{}$” than “$\varphi_{f}^{}$” is obtained in PCNT containing interphase, due to the confident effect of interphase in the effective particles. So, a thick interphase, which is obtained by strong interfacial bonding increases the effectiveness of nanoparticles in PCNT and promotes the properties of nanocomposite. The good impacts of thick interphase on the mechanical behavior of polymer nanocomposites were also observed in the literature [38,61]. The ineffective role of “u” parameter in “$\varphi_{eff}^{}$” term (Figure 2b) may be due to the more significant level of equivalent length than “R” and “t” (Equation (4)). Accordingly, the “$\varphi_{eff}^{}$” parameter only depends on “R” and “t” parameters and the length and waviness of CNT do not change its level.

**Figure 3:**
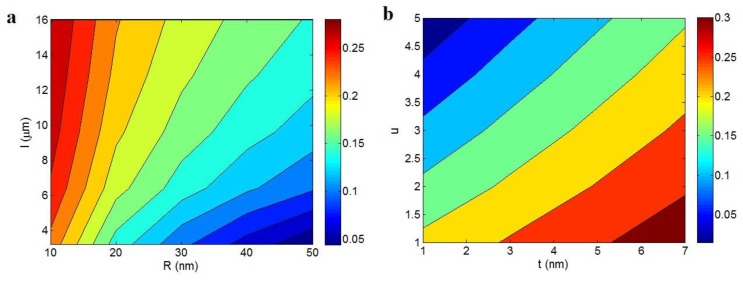
The variations of “f_iu_” term at different levels of (**a**) “R” and “l” and (**b**) “t” and “u” parameters according to Equation (6).

Figure 3 exhibits the effects of different parameters on “f_iu_” term as the fraction of percolated filler above percolation threshold using Equation (6). Figure 3a indicates that the least “f_iu_” as about 0.05 is obtained at R = 50 nm and l = 3 μm, while the smallest level of “R” and l > 10 μm significantly improve the level of “f_iu_” to 0.28. So, the highest fraction of percolated filler is obtained by the thinnest and the largest CNT based on the networkability of thin and large CNT. In other words, the potential connection and formation of a filler network in thin and long CNT are more than thick and short ones, because they can easily find each other in PCNT. It means that the large aspect ratio of these nanoparticles causes low distance and more Head-Head and Head–Tail interaction/contact between nanotubes, which facilitate their networking. However, the random distribution of thick and short CNT in PCNT makes a high distance among nanotubes, which cannot promote the networking. 

Figure 3b also observes the roles of “t” and “u” parameters on “f_iu_” ranges. The highest outputs are acquired by the high level of “t” and low “u”, where f_iu_ = 0.3 is estimated at t > 6 nm and u < 1.5. However, a low “t” and great “u” result in a poor “f_iu_” demonstrating that the interphase thickness and waviness show different effects on the fraction of percolated CNT in PCNT. A high “f_iu_” is obtained by thick interphase and low waviness, whereas the thin interphase and high waviness cannot improve the “f_iu_” level. The thick interphase surrounding CNT can persuade the separated CNT with high segregation distance to connection and networking. In fact, a thick interphase approaches the nanotubes and increases the possibility of networking. As a result, the number of CNT in network phase as “f_iu_” grows by a thick interphase. Obviously, a thin interphase cannot play an effective role in PCNT, which negligibly changes “f_iu_”. On the other hand, the negative role of “u” parameter in “f_iu_” is attributed to the reduced length of CNT, which decreases the effective interaction among CNT for connection. Undoubtedly, more waved CNT (high u) produce a high level of segregation distance between nanotubes in PCNT, which decreases the probability of networking. Accordingly, the waved CNT negatively affected the joining of nanotubes and production of a dense network, which is represented here by their negative role in “f_iu_” term. 

**Figure 4:**
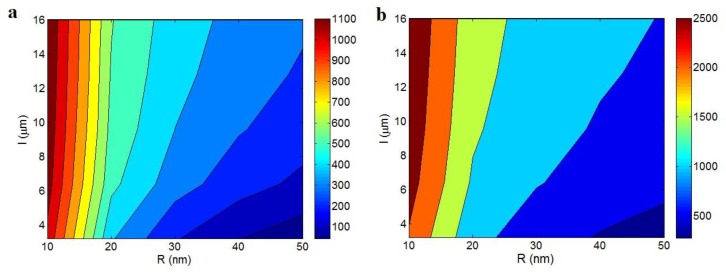
The roles of “R” and “l” parameters in the electrical conductivity of PCNT based on (**a**) conventional equation (Equation (9) at b = 2 and (**b**) developed model (Equation (12)).

Figure 4 illustrates the influences of “R” and “l” parameters on the electrical conductivity of PCNT predicted by the conventional and developed models in Equations (9) and (12). Figure 4a indicates that the least “R” and the high levels of “l” produce the highest conductivity. The “σ” level of 1100 S/m is observed at R = 10 nm an l > 6 μm. However, the conductivity significantly decreases at high and small levels of “R” and “l” parameters, respectively. Conclusively, the thinner and longer CNT produce a better conductivity in PCNT. Figure 4b also represents the similar trends between conductivity and these parameters based on the developed model, but the developed model predicts a higher level of conductivity at same levels of “R” and “l”.

The high values of radius and length of CNT cause negative and positive effects on the conductivity of nanocomposites. In general, the CNT significantly increase the poor conductivity of polymer matrix, which is in the range of 10^−12^- 10^−15^ S/m. However, the thin and large CNT quicken the formation of filler network in PCNT, increase the effective volume fraction of CNT and also enhance the fraction of percolated CNT. So, the thin and long CNT, which occupy a large region of PCNT promptly produce a conductive network in PCNT at a very low percolation threshold and then, produce a big network in PCNT, which can effectively transport the electrons whole of PCNT and produce a significant electrical current. Therefore, the conduction efficiency of CNT in PCNT improves more by thinner and larger nanoparticles, as reported by the experimental measurements of electrical conductivity [62,63]. 

**Figure 5:**
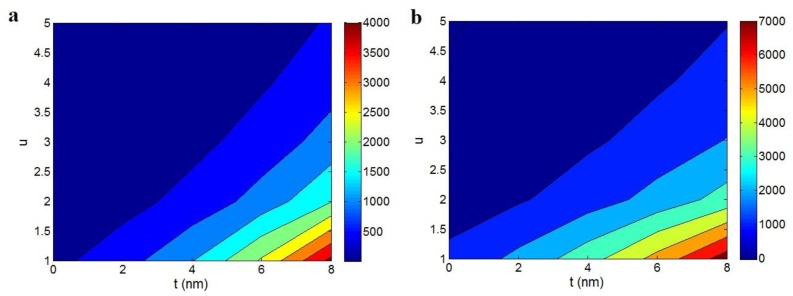
The electrical conductivity of PCNT at different “t” and “u” values according to (**a**) conventional model (b = 2, Equation (9)) and (**b**) developed equation (Equation (12)).

Figure 5 reports the calculations of electrical conductivity at different levels of “t” and “u” parameters by the suggested models. Both models show the highest conductivity at the highest and the smallest levels of “t” and “u” factors, respectively. The conventional model (Figure 5a) calculates the conductivity of 4000 S/m at t = 8 nm and u = 1, while conductivity decreases to about 0 at low levels of “t” and high values of “u”. Moreover, Figure 5b based on the developed model demonstrates the highest conductivity as 7000 S/m at t = 8 nm and u = 1, while an insulated nanocomposite is approximately observed at the low and great values of “t” and “u” parameters, respectively. As a result, both models theoretically indicate that the conductivity more improves by thicker interphase and less waviness. On the other hand, the conductivity does not increase in PCNT containing thin interphase and waved CNT. In conclusion, the interphase thickness and the waviness of CNT directly and inversely affect the electrical conductivity of PCNT.

The high efficiencies of a thick interphase in percolation threshold, effective CNT and percolated nanotubes were mentioned. Now, it can be stated that the thick interphase supports the nanoparticles to form a conductive network at a low percolation. Furthermore, it is theoretically considered that a thick interphase pushes the nanotubes to form a big network in PCNT by connecting the interphase regions. All these phenomena promote the charge transfer in PCNT, which improves the electrical conductivity of PCNT. In fact, the formation of interphase layer around the nanoparticles as tunneling distance undoubtedly enhances the conducting efficiency of CNT in PCNT. Previous articles reported the tunneling conduction in the nanocomposites [64,65,66]. This phenomenon can be verified by plotting current vs. voltage in the nanocomposite, which exhibited a highly non-linear behavior [66].

Previously, the reinforcing effect of interphase regions in polymer nanocomposites was well reported in the articles [61,67]. In addition, u = 1 produces the highest conductivity in PCNT, because it demonstrates the straight CNT without waviness (Equation (2)). The negative role of waviness in the conductivity of PCNT is interpreted by its impact on the effective length and natural conductivity of CNT. The waved CNT produce a short length, which worsens the percolation threshold for network formation and the fraction of percolated nanoparticles, which both result in less effective CNT. These remarks indicate that the waviness of CNT postpones the formation of conductive structure in PCNT and also, decreases the size and density of network. Accordingly, experimental and theoretical data indicate that the waviness of CNT losses the effectiveness of the CNT network for electron transferring, which leads to a poor electrical conductivity in PCNT. On the other hand, the natural conductivity of CNT weakens by their waviness as illustrated in Equation (8). So, the networks of waved CNT show less conductivity compared to those containing straight nanotubes, which cannot significantly improve the electrical conductivity of insulate polymer matrices.

**Figure 6:**
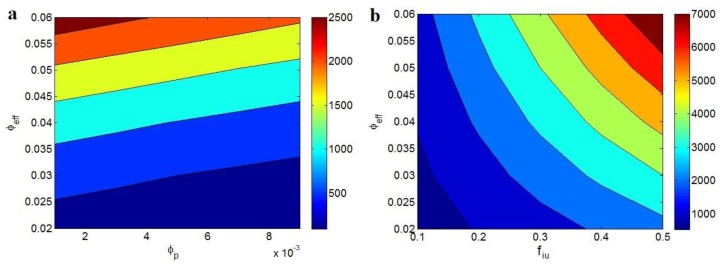
The variations of electrical conductivity in PCNT by (**a**) various levels of “$\varphi_{p}^{}$” and “$\varphi_{eff}^{}$ ” parameters at b = 2 by conventional model (Equation (9)) and (**b**) different “f_iu_” and “$\varphi_{eff}^{}$ ” ranges based on the developed model (Equation (12)).

The effects of “$\varphi_{p}^{}$” and “$\varphi_{eff}^{}$” parameters on the conductivity of PCNT predicted by conventional model are depicted in Figure 6a. A low value of “$\varphi_{eff}^{}$” decreases the conductivity to about 0, but the smallest value of “$\varphi_{p}^{}$” and the highest level of “$\varphi_{eff}^{}$” produce the highest conductivity as 2500 S/m. Therefore, the least percolation threshold and the highest effective CNT fraction increase the conductivity of PCNT. Moreover, Figure 6b demonstrates that the high levels of both “f_iu_” and “$\varphi_{eff}^{}$” parameters positively improve the conductivity of PCNT based on the developed model. The conductivity of 7000 S/m is observed at f_iu_ = 0.5 and $\varphi_{eff}^{}$ = 0.06, while the poor conductivity of about 700 S/m is estimated at f_iu_ = 0.1 and $\varphi_{eff}^{}$ = 0.02. Therefore, the low value of “$\varphi_{p}^{}$” as well as the great ranges of “f_iu_” and “$\varphi_{eff}^{}$” parameters causes the high conductivity of PCNT. 

The percolation threshold is the critical volume fraction of CNT in PCNT in which the conductive network forms. Accordingly, its low level produces the conductivity in PCNT at very low concentration of CNT. In addition, the helpful impact of low “$\varphi_{p}^{}$” on “f_iu_” is understood from Equation (6), which suggests that a low percolation threshold produces a percolated network by a large number of CNT. According to these explanations, the promotion of conductivity by small percolation level is unavoidable. Moreover, a high level of “$\varphi_{eff}^{}$” expresses that the CNT dimensions and interphase thickness produce more effective CNT, which considerably grows the electrical conductivity of PCNT. Furthermore, a great level of “f_iu_” shows the involvement of a large quantity of CNT in the percolated phase producing large and dense networks in PCNT, which can quickly transport the charge in PCNT. Accordingly, a higher “f_iu_” is representative of a bigger and denser network of CNT in PCNT, which significantly stimulates the electrical conductivity.

## 4. Conclusions

The effective volume fraction of CNT, percentages of percolated filler after percolation threshold and electrical conductivity in PCNT were expressed assuming the tunneling effect by interphase layer around nanoparticles. Thin and long CNT, thick interphase, and low waviness caused positive effects on the fraction of percolated CNT, but a high effective filler fraction was only obtained by thin CNT and thick interphase. Thin and long CNT caused a low distance among nanotubes in PCNT, which increased the probability of connection and networking. Also, a thick interphase showed a strong contribution to the connection of CNT, because it can push the separated nanotubes to form a conductive network. The waviness also decreased the effective length of nanotubes, which weakened their percolation and joining. According to these explanations, it was logical to obtain the highest conductivity of PCNT by the thinnest and the longest CNT, the thickest interphase, and the smallest waviness. These parameters effectively changed the effective properties of CNT and network; so, they significantly governed the electrical conductivity of PCNT. It was also reported that a low value of “$\varphi_{eff}^{}$” did not change the conductivity of PCNT, but the smallest $\varphi_{p}^{}$= 0.001 and the highest $\varphi_{eff}^{}$ = 0.06 produced the highest conductivity of 2500 S/m by conventional model. Also, the highest conductivity of 7000 S/m was observed at the highest values of f_iu_ = 0.5 and $\varphi_{eff}^{}$ = 0.06 based on the developed model. These evidences demonstrated that the low value of “$\varphi_{p}^{}$” as well as the great ranges of both “f_iu_” and “$\varphi_{eff}^{}$” terms induced the high conductivity of PCNT.
